# Breaking the gatekeepers: how AI will revolutionize scientific funding

**DOI:** 10.3389/frai.2025.1667752

**Published:** 2025-08-29

**Authors:** Madhur Mangalam

**Affiliations:** Department of Biomechanics, University of Nebraska at Omaha, Omaha, NE, United States

**Keywords:** peer review, research evaluation, early-career scientists, research funding, grant application

## Abstract

As artificial intelligence (AI) transforms nearly every domain of human endeavor, one of its most consequential impacts may be on science itself. This analysis explores how AI technologies could disrupt the power structures that govern research funding—structures that privilege senior investigators while sidelining early-career scientists and genuinely novel ideas. By juxtaposing the youth-driven innovation behind AI with the increasingly gerontocratic funding patterns in biomedical sciences, we highlight how institutional mechanisms shape not only who gets to do science but also *when*. Evidence suggests that conventional grant peer review has become a self-reinforcing system—more effective at preserving consensus than fostering discovery. AI presents a compelling alternative: evaluation frameworks that could reduce bias, broaden participation, and open more meritocratic pathways to research independence. The implications extend far beyond individual careers. At stake is society's ability to mobilize scientific creativity against its most urgent challenges. By rethinking outdated practices—especially the gatekeeping role of study sections—and exploring algorithmic approaches to assessment, we may be able to reverse troubling trends and unleash a broader, more diverse wave of discovery. AI will not fix science on its own, but it could help build a system where innovation is no longer an accident of privilege and timing.

## 1 Structural problems in scientific funding

Contemporary scientific funding systems exhibit persistent and well-documented structural biases that disproportionately disadvantage early-career researchers, while simultaneously favoring established investigators engaged in safe, incremental lines of inquiry. The average age of first-time NIH grant recipients has increased dramatically over the past few decades, with researchers under 40 receiving only 29% of R01 grants in 2010, compared to 43% in 1980 (Rockey, 2012). This shift coincides with mounting evidence of reviewer bias against novel or high-risk research approaches, where innovative proposals routinely receive lower scores than equally rigorous but more conventional submissions (Boudreau et al., 2016).

As Alberts et al. (2014) warned, the system now privileges certainty over creativity, experience over originality. The consequence is structural: a persistent tilt toward incrementalism and a quiet exile of the next generation of innovators. When bold ideas are consistently filtered out in favor of safe bets, the pace of discovery slows. While many of history's most transformative scientific breakthroughs were made by researchers in their twenties and thirties, recent decades have seen a marked institutional shift: the peak age of great achievement has risen from around 30 in 1900 to nearly 40 in 2000, largely driven by prolonged training, delayed independence, and structural disincentives for early-career innovation (Jones, 2010).

The authors argue that the long-standing assumption of continuous growth in biomedical research has led to a system overloaded with scientists competing for a shrinking pool of resources. This has created a hyper-competitive environment that discourages new talent, overloads experienced researchers, and misaligns the training pipeline with available career opportunities (Alberts et al., 2014). Innovative ideas without institutional backing are often overlooked, as merit is frequently conflated with visibility rather than substance.

This risk aversion has emerged alongside striking demographic changes in federal research funding recipients. For instance, in 1998, early-stage investigators (ages 24–40) and established investigators (ages 56 and older) each comprised about 20% of NHLBI grant recipients, with mid-career investigators (ages 41–55) making up the remaining 60%. By 2014, the proportion of early-stage awardees had stabilized at a lower level, while the proportion of established investigators had risen linearly and surpassed that of early-stage awardees—signaling a demographic shift toward an older funded population (Charette et al., 2016). This reflects the institutional ossification of a funding system that systematically undervalues early-career researchers, regardless of their creativity, technical sophistication, or transformative potential.

The consequences extend beyond individual careers to affect the pace and direction of scientific progress. As Azoulay et al. (2011) argue, most current funding mechanisms are explicitly designed to reward safe proposals, discourage experimentation, and enforce short-term deliverables. The result is a system that routinely confuses risk with recklessness and treats innovation as a form of impertinence rather than ambition.

At the core of this conservatism is the study section itself—a peer review mechanism that has morphed from a panel of equals into a high-stakes citadel of professional self-preservation. Li and Agha (2015) show that reviewers tend to favor applicants from their own academic networks, theoretical orientations, and methodological schools. This intellectual and social insider effect locks in epistemic homogeneity and blocks the entry of novel perspectives. In a landmark study, Boudreau et al. (2016) found that even when two proposals are of equal quality, reviewers consistently give lower scores to the more novel one, with bias magnitude sufficient to fully offset the novelty premium. Increasingly, study sections demand extensive pilot data as a condition of funding, creating a Catch-22 where researchers need funding to generate the very preliminary results required to justify funding (Alberts et al., 2014).

Travis and Collins (1991) document how study sections are composed of researchers who often have overlapping training histories, co-authorships, and institutional affiliations. This creates recursive vetting by an insider class rather than genuine peer review. The cumulative result is what sociologist Robert Merton famously termed the Matthew effect (Merton, 1968)—a systemic pattern where scientific recognition, resources, and visibility accrue to those who already possess them.

Despite their central role in allocating billions of research dollars, there is remarkably little empirical evidence that study sections consistently succeed at identifying the most impactful proposals. As Smith (2006) and others have shown, review outcomes are often influenced more by panel composition and subjective dynamics than by the intrinsic quality of the science. In a landmark evaluation, Jacob and Lefgren (2011) compared the publication trajectories NIH applications that scored just above and just below the funding cutoff, revealing no significant differences in subsequent scientific output between the two groups.

The history of science includes numerous transformative breakthroughs that were initially dismissed by institutional gatekeepers. Kary Mullis' early proposal for the polymerase chain reaction (PCR) was denied NIH support (Mullis, 1990). Hans Krebs' elucidation of the citric acid cycle was rejected by *Nature* before earning him a Nobel Prize (Krebs and Johnson, 1980). Barry Marshall's discovery that *Helicobacter pylori* causes ulcers faced years of funding rejection before transforming clinical medicine (Marshall and Warren, 1984). Campanario (2009) systematically cataloged 24 cases where Nobel-winning papers were initially rejected by peer reviewers.

The crisis of evaluation is compounded by the economics of academic labor. As institutions increasingly shift to soft money models where salaries are contingent on extramural grants, established investigators face continuous existential pressure to maintain funding. These same individuals disproportionately serve on review panels, voting on proposals that draw from the very pool of resources they themselves must compete for to survive.

## 2 AI research as a counter-example of innovation

While the biological sciences have grown increasingly dominated by researchers in their fifties and sixties—many of whom did their most innovative work decades earlier—artificial intelligence presents a striking counter-example. It is a field where early-career scientists not only contribute but routinely lead transformative breakthroughs. This divergence is not due to differences in intellectual complexity, experimental rigor, or the maturity of the disciplines themselves. Rather, it reflects how institutional design and cultural norms determine who may innovate, when that opportunity is granted, and under what conditions merit matters. The lesson is clear: scientific vitality is not merely a function of content, but of context.

This is not just theoretical—the recent history of AI is filled with early-career breakthroughs that illustrate the point. In 2012, Alex Krizhevsky, then a graduate student, led the development of AlexNet—the deep learning system that revolutionized computer vision and launched the modern AI boom (Krizhevsky et al., 2012). In 2014, Goodfellow et al., while still a PhD student, invented Generative Adversarial Networks, a technique now foundational in image generation and media synthesis (Goodfellow et al., 2014). In 2017, Vaswani et al., as a relatively early-career research scientist, was the first author on the paper introducing the Transformer architecture—the foundation of nearly all large language models today (Vaswani et al., 2017). In 2019, Devlin et al. developed BERT, a pretraining method for natural language processing that continues to shape both industrial and academic research (Devlin et al., 2019).

What makes artificial intelligence so conducive to early-career innovation? The answer lies not in the technical content of the field, but in its surrounding structures—its infrastructure, its incentives, and its evaluative norms. These factors make AI a uniquely fertile ground for merit-based disruption by those outside traditional power hierarchies. Unlike experimental sciences that require multi-million-dollar facilities, complex instrumentation, and long institutional lead times, AI research is primarily computational. While the most advanced models require enormous resources, many key innovations—including AlexNet and BERT—began with comparatively modest infrastructure (Ahmed and Wahed, 2020). Open-source libraries such as PyTorch and TensorFlow provide powerful toolkits freely available to anyone with internet access and technical fluency. Public datasets enable rigorous benchmarking without expensive data collection, and cloud computing reduces the barrier to running experiments at scale. This accessibility creates a technical environment where a determined graduate student can meaningfully contribute to the state of the art without institutional gatekeeping.

AI researchers have benefited from research cycles that are orders of magnitude faster than in the biological sciences. Unlike lab experiments that can take months to prepare and even longer to interpret, AI experiments can often be implemented, trained, and evaluated in hours or days. Zoph and Le (2016) document automated architecture search procedures that evaluate thousands of model variants within days. These rapid feedback loops enable empirically grounded learning through high-throughput experimentation, allow researchers to build intuition through direct, iterative refinement, reduce the sunk cost of failure thereby de-risking intellectual exploration, and empower students to test unconventional ideas without formal institutional approval. These dynamics particularly benefit early-career researchers, who gain traction through experimentation, not slow institutional ascent or apprenticeship. They also invert the typical power structure of slow-moving fields, where access to equipment and mentoring is a precondition for experimentation. In fast-feedback environments, ideas compete before résumés do.

Equally important, AI researchers operate in an ecosystem supported by multiple funding channels beyond traditional academic grants (Arora et al., 2020). These include venture capital for AI startups and research commercialization, corporate research labs with autonomy and experimental freedom, industry fellowships and open-access research grants, non-traditional philanthropic or advocacy-backed initiatives, and open-source community collaborations and support. Unlike biomedical research—where approximately 80% of academic funding comes through the NIH, a single, centralized gatekeeper (Moses et al., 2015)—AI researchers can pursue multiple avenues. A student with a promising idea can seek VC backing, publish via open platforms, or contribute to a community-led project without first winning institutional endorsement. This pluralism in funding pathways unlocks the possibility of meaningful innovation by those whose ideas may be unproven, unpopular, or considered premature by conventional academic or institutional standards. It also helps reduce the disciplinary and professional cost of failure, allowing researchers to take intellectual and methodological risks without jeopardizing their entire career trajectory.

AI culture prizes speed, openness, and reproducibility over gatekeeping and credentialism. Preprint servers like arXiv enable immediate dissemination of findings, bypassing the months-long delays and status-dependent bottlenecks of peer-reviewed journals (Soergel et al., 2013). Research is judged in the open, and often implemented in production systems within weeks, regardless of the author's rank or affiliation. A graduate student can post a novel architecture, and if it performs well, it may be integrated into commercial applications before they defend their dissertation. The open publication culture in AI research means an idea's influence is determined by its inherent quality and utility—not by the journal's status or the author's seniority. The culture does not eliminate hierarchy—but it does allow work to speak for itself. In doing so, it offers early-career researchers a path to visibility and influence untethered from traditional credentials.

These cultural and infrastructural conditions form a uniquely generative and dynamic ecology for early-career researchers. They enable ideas to gain visibility through demonstrated performance rather than proximity to institutional power. As a result, AI remains one of the rare scientific fields where breakthroughs emerge not despite youth, but often because of it. The contrast with biomedical sciences could not be more dramatic. Where AI rewards speed, openness, and pluralism, biomedical research remains governed by centralized review, extended timelines, and vertically stratified hierarchies. The institutional structure of scientific funding profoundly shapes who can innovate, when they can innovate, and what ideas receive resources (Azoulay et al., 2011). Where AI creates paths for merit to rise on its own terms, biology often demands that it wait its turn.

In short, the aging of scientific innovation is not a natural function of intellectual maturity, disciplinary complexity, or the inherent difficulty of modern problems. It is the artifact of institutional architecture—systems that delay, dilute, or deny opportunities for boldness, depending on who holds the keys and how tightly they guard them. More importantly, it suggests an alternative: that by applying the cultural principles and structural affordances that have made AI fertile ground for early-career innovation, other scientific fields could begin to reclaim their futures—not by lowering standards, but by removing the barriers that prevent boldness from taking root.

## 3 The transformative potential of AI in research evaluation

What if the technologies driving the AI revolution could be applied not only to scientific discovery itself, but also to the very systems that determine which discoveries are allowed to happen in the first place? AI offers profound and timely potential for rethinking how we allocate scientific resources—particularly in confronting the structural limitations, entrenched hierarchies, and well-documented biases of human review panels. Its promise lies not in merely automating existing procedures, but in enabling new forms of evaluation grounded in transparency, scalability, and epistemic diversity. AI could help construct more equitable, inclusive, and forward-looking pathways to research funding—pathways that prioritize intellectual merit over institutional prestige. At stake is not just the efficiency of evaluation, but the architecture of access, legitimacy, and innovation in science itself.

### 3.1 Detecting novelty and impact potential

Traditional peer review has consistently shown bias against research that breaks from conventional paradigms, favoring incremental advances over ambitious leaps (Boudreau et al., 2016). AI systems, by contrast, can evaluate proposals not through the narrow lens of disciplinary conservatism, but through patterns of innovation embedded across the entire scientific landscape. Natural language processing can assess the semantic content of proposals against vast corpora of scientific literature, identifying novel concept combinations and underexplored intersections (Wang et al., 2013). Unlike human reviewers bounded by personal expertise, AI can systematically scan the full terrain of knowledge. Machine learning systems can detect linguistic and structural signals that have historically preceded transformative discoveries. If certain semantic and stylistic markers are reliably associated with breakthroughs, and AI can recognize them without institutional bias, it may help surface transformative ideas that would otherwise be overlooked. Graph-based learning can simulate the potential diffusion of proposed ideas through the scientific ecosystem. Zeng et al. (2017) demonstrated that these models can outperform expert judgment in predicting future citation impact and field-level influence.

These techniques could help identify the very kinds of proposals—like those for PCR or *Helicobacter pylori*—that traditional review processes have historically rejected, only to see them later revolutionize their fields. Because AI can operate across fields, detect subtle signals, and remain agnostic to disciplinary prestige, it may be uniquely positioned to recognize the value of ideas before institutions do. AI could help shift the system from protecting the status quo to discovering what lies beyond it.

### 3.2 Reducing human bias through algorithmic fairness

Human review panels exhibit persistent biases related to race, gender, institutional prestige, and methodological orthodoxy (Ginther et al., 2011; Hofstra et al., 2020; Li and Agha, 2015; Witteman et al., 2019). When designed with care, transparency, and accountability, AI systems offer powerful mechanisms for dismantling entrenched inequities. AI can enforce stricter separation between applicant identity and proposal content, focusing attention on ideas rather than pedigree. Unlike human reviewers, who unconsciously infer prestige from names, institutions, or writing style, AI can be deliberately blinded to such cues. Fairness-aware algorithms can be explicitly designed to audit and adjust for disparities across demographic and institutional dimensions. As Kleinberg et al. (2018) note, algorithmic systems can be tuned iteratively to improve parity, while human committees rarely correct for their biases, even when known. AI systems can be continuously monitored and retrained based on *post-hoc* outcome data. Unlike fixed human committees, algorithms can evolve in response to bias audits, error analysis, and real-world disparities.

This explicitness makes it possible to systematically measure both the intended and unintended consequences of different designs. This stands in stark contrast to the implicit, often opaque processes of human judgment, where evaluative criteria tend to be diffuse, unstandardized, and largely shielded from meaningful scrutiny. Beyond questions of fairness, AI systems can broaden what review panels even recognize or consider as valid evidence of merit.

### 3.3 Expanding the information base for evaluation

Traditional peer review has historically relied on a narrow and fragmented stream of information: the written proposal, the applicant's CV, and the limited, often idiosyncratic knowledge that a small group of reviewers happens to bring to the table. By contrast, AI systems can dramatically expand the evaluative horizon through continuous, data-rich integration of diverse sources that no human committee could feasibly synthesize. AI models can synthesize publication history, citation dynamics, data sharing practices, software reproducibility, prior funding efficiency, and mentorship outcomes into a multidimensional assessment (Fortunato et al., 2018). These factors can be weighted and calibrated for context, offering a fuller picture than reputation or impact factor alone. Machine learning algorithms can continuously scan, analyze, and map emergent research areas, enabling reviewers to assess alignment with fast-evolving scientific frontiers rather than rely on outdated assumptions or legacy paradigms. While human reviewers are often years behind the bleeding edge of innovation, AI can operate in near real time, adapting as new knowledge surfaces. Many significant breakthroughs originate precisely at the edges and intersections between disciplines. AI systems trained on literature across domains can detect transdisciplinary relevance and assess impact in areas reviewers may overlook (Foster et al., 2015). This helps address one of peer review's most stubborn blind spots: its bias against boundary-crossing science.

This broadened evaluative base would allow more accurate assessment of early-career researchers, interdisciplinary thinkers, and novel approaches that traditional panels may undervalue. These capabilities do not aim to replace expert judgment—they aim to extend it with the breadth, speed, and self-correcting capacity that only machine intelligence can provide. Of course, these systems must be built and audited with the same transparency and fairness they aim to enforce.

### 3.4 Potential applications: AI in research funding

Though still in early phases of implementation, the application of AI in research funding represents a rapidly emerging frontier. Conceptual models and emerging pilot programs illustrate how AI could not only augment existing evaluation processes but also help surface overlooked ideas, reduce systemic bias, and enhance transparency. These potential applications offer more than technical reform—they suggest how AI might reshape not just the mechanics, but the institutional culture of scientific funding.

### 3.5 Potential AI-enhanced evaluation systems

AI platforms could be designed to identify high-risk, high-reward proposals that might be overlooked by conventional peer review. Such systems could employ natural language processing to analyze proposal content in relation to the broader scientific literature. They might be able to flag promising, unconventional proposals that would otherwise receive low ratings from human reviewers. These models could be especially effective in elevating proposals from early-career researchers and applicants outside traditional institutional power centers.

Algorithmic approaches to proposal evaluation could potentially identify innovative research that challenges existing paradigms—precisely the type of work human reviewers tend to undervalue (Fortunato et al., 2018). Such systems could act as amplifiers of intellectual diversity—especially where entrenched review cultures tend to filter it out. By systematically surfacing overlooked potential, AI systems could help correct for the structural conservatism that has long skewed scientific resource allocation.

### 3.6 Machine learning for reducing evaluation bias

Future machine learning approaches could be designed to augment the first-pass screening of grant applications. Models could be trained on historical funding decisions while explicitly correcting for known demographic and institutional biases. Such systems could potentially match human reviewers in predictive accuracy while significantly reducing bias in scoring. Automating initial screening could enable reviewers to devote more time and scrutiny to borderline cases that require genuine deliberation.

The systematic nature of algorithmic evaluation creates an opportunity to explicitly correct for known biases in ways that *ad hoc* human judgment cannot. Rather than replacing peer review, AI could be deployed to rebalance it—helping funding institutions uphold commitments to equity without compromising scientific quality, provided the systems are implemented with transparency and rigorous auditing.

### 3.7 Algorithmic approaches to reviewer selection

AI systems might improve equity and innovation in reviewer selection itself. Such systems could be designed specifically to identify reviewers with diverse perspectives and expertise beyond traditional metrics. This approach might lead to funding more diverse applicants across dimensions of geography, career stage, and institutional prestige. Theoretically, such diversity in review could help identify proposals with greater innovation potential.

Diversifying reviewer backgrounds and perspectives could fundamentally alter which proposals receive support, potentially favoring more innovative approaches (Li and Agha, 2015). Such systems would not merely aim to streamline peer review—they would challenge its epistemic foundations and rebuild them around broader principles of inclusion and innovation. By reconfiguring the architecture of peer review itself, algorithmic reviewer selection could help dismantle entrenched networks of epistemic authority and expand the voices long excluded from gatekeeping roles.

Taken together, these use cases point toward a future in which AI does not merely assist review but redefines what scientific promise looks like—and who gets to define it.

These emerging applications suggest that AI could do more than replicate the logic of traditional peer review—it could expose its blind spots and offer a blueprint for its reinvention. By broadening the definition of merit, dampening the persistent signal of bias, and creating new pathways for unconventional voices to be heard, AI-assisted review systems may help surface precisely the kinds of science that entrenched structures are least equipped to recognize. Though still in development, these approaches point toward a future in which evaluation is not only more efficient but also more equitable, inclusive, and aligned with the true spirit of scientific inquiry.

### 3.8 Implementation pathways: how AI could transform scientific funding

The most promising near-term strategy for integrating artificial intelligence into research funding lies not in full automation, but in carefully designed hybrid systems that combine algorithmic insight with human oversight at each stage of the evaluation process. Such systems could preserve the ethical reasoning, contextual awareness, and domain-specific insight of human judgment while leveraging the consistency, scalability, and pattern recognition strengths of machine learning. If implemented thoughtfully and transparently, these hybrid models could serve not only as agents for efficiency but as scaffolds for building a more accountable, inclusive, and innovation-oriented funding ecosystem (Table 1). While current AI systems remain limited in their interpretability and domain transferability, their evaluative potential continues to grow—particularly in hybrid frameworks with careful constraints. Designing such systems today is not just a technical challenge, but a moral imperative—an opportunity to rebuild evaluation infrastructures before they entrench further the very inequalities they ought to correct.

**Table 1:** Comparison of traditional peer review and AI-hybrid funding evaluation systems.

**Current peer review system**	**AI-hybrid evaluation pathway**
Heavily reliant on human judgment, often influenced by cognitive bias and professional networks.	Combines algorithmic triage and bias detection with human expertise for more balanced decision-making.
Emphasizes prior track record and institutional prestige.	Prioritizes proposal quality, novelty, and potential impact, independent of credentials or affiliation.
Slow, opaque, and labor-intensive evaluation processes.	Faster, more transparent screening through automated systems, enabling broader and more inclusive applicant pools.
Focus on identifying flaws and eliminating risk.	Designed to identify promise, support risk-taking, and explicitly allocate funding to high-risk/high-reward research.
Limited feedback loops or performance tracking.	Enables continuous outcome monitoring, feedback, and system refinement based on real-world evidence.
Tends to reinforce existing disciplinary hierarchies and funding patterns.	Encourages methodological, demographic, and institutional diversity through portfolio optimization.

One practical pathway involves a tiered review system that distributes evaluative labor across complementary stages. Initial AI screening through algorithmic analysis could identify promising proposals, with a focus on novelty, interdisciplinarity, and potential impact. This step would counteract the documented tendency of human reviewers to undervalue unorthodox or paradigm-challenging research (Boudreau et al., 2016). Following this automated screening, blind human review by reviewers selected to minimize conflicts of interest would evaluate a subset of anonymized proposals flagged by the algorithm. By decoupling reviewer identity from applicant credentials, this step could significantly reduce homophily, prestige bias, and gatekeeping effects. Finally, during AI-assisted panel discussions for final decision-making, AI systems could provide real-time analysis of reviewer behavior, flag potential inconsistencies, and surface latent bias patterns in scoring or commentary. These AI applications would function not as judges, but as mirrors—making institutional blind spots visible precisely when they matter most, during high-stakes decisions about funding and recognition. Such a system would not only streamline evaluation processes, but also help make them fairer, more transparent, more consistent, and more capable of identifying scientific ideas that challenge the status quo. In doing so, it could begin to shift the culture of peer review itself—from subjective gatekeeping to a more structured, evidence-based, and accountable mode of deliberation.

While AI-enhanced evaluation systems offer significant promise, it is equally important to acknowledge that traditional peer review still performs critical functions that must be preserved and integrated. Human reviewers bring irreplaceable domain expertise, contextual understanding of field-specific nuances, and the capacity to assess research ethics, feasibility, and broader scholarly relevance in ways that current AI systems cannot yet fully replicate. The collegial aspects of peer review—including mentorship, community building, and the transmission of disciplinary standards—represent valuable social functions beyond mere evaluation. Moreover, AI systems face inherent limitations including potential algorithmic bias amplification if training data reflects historical inequities, difficulties in evaluating truly interdisciplinary or paradigm-shifting research that lacks historical precedent, and challenges in assessing subjective elements like research elegance, theoretical sophistication, or investigator resilience. Any implementation of AI-enhanced evaluation must therefore be designed as a complement to, rather than replacement for, human expertise, preserving the collaborative and mentoring dimensions of scientific review while addressing its documented biases and structural limitations.

Beyond evaluating individual proposals in isolation, AI could be harnessed to optimize entire funding portfolios across multiple dimensions of scientific value and risk. Through portfolio optimization algorithms, funders could explicitly balance exploratory and incremental projects by allocating resources according to risk profiles—ensuring that a dedicated portion supports high-risk, high-reward research that might otherwise be excluded by conservative review processes (Boudreau et al., 2016). AI systems could also help ensure equitable representation across career stages. As Jones and Weinberg (2011) demonstrate, early-career researchers are more likely to pursue novel ideas, while senior researchers contribute depth and continuity—both essential to a thriving scientific ecosystem. Similarly, portfolio strategies could promote methodological diversity, supporting varied approaches to the same research problem as a hedge against epistemic blind spots. Scientific breakthroughs often arise not from consensus methods but from methodological outsiders (Foster et al., 2015). Critically, portfolio-based strategies have been shown to outperform project-by-project evaluations in maximizing long-term scientific progress (Wang et al., 2013). At scale, AI can make these strategies tractable—dynamically adjusting funding distributions to optimize discovery across disciplines, time horizons, and theoretical frameworks. Such a shift would enable funding agencies to move from reactive gatekeeping toward proactive, ecosystem-level stewardship of science.

Despite their promise, AI-based evaluation systems pose serious risks that must be confronted through thoughtful design, transparent governance, and public accountability. Models trained on historical funding decisions risk encoding and perpetuating inequities, as biased training data may reflect structural exclusions embedded in past review outcomes. To mitigate these harms, strategies such as reweighting for underrepresented applicants, incorporating explicit novelty or risk-taking bonuses, and generating synthetic training datasets that break from legacy patterns should be prioritized. Moreover, opaque or black-box evaluations will fail to earn the trust of researchers and institutions alike. Transparency and explainability are not optional—they are foundational to legitimacy. Explainable AI techniques, open-source evaluation criteria, and structured appeals processes are essential for ensuring procedural fairness and due process. Crucially, research funding is not merely a matter of technical merit; it reflects societal priorities and normative values. Safeguarding those values requires democratic oversight, value-aligned model objectives, and integration with ethical review structures to ensure that algorithmic methods reflect the human stakes of scientific judgment. Failure to meet these ethical and technical challenges risks entrenching the very injustices that AI is often invoked to remedy. But with proactive safeguards, AI systems can serve not only as instruments of efficiency but as mechanisms for epistemic integrity and institutional repair.

Taken together, these implementation pathways offer not just operational reform but a bold new epistemic blueprint for how science allocates trust, risk, and opportunity. One in which human expertise and algorithmic automation collaborate rather than compete; one in which merit is thoughtfully disentangled from prestige and inherited privilege; and one in which bold, uncredentialed ideas are not prematurely filtered out at the start. It is a vision of evaluation as infrastructure for discovery—not as a filter for conformity, but as a scaffold for possibility. AI will not eliminate human bias, but it can help us name it, mitigate it, and—where necessary—route around it. If designed with humility, transparency, and ethical resolve, AI-enabled funding systems could help re-engineer science's most powerful lever: the ability to decide what gets discovered, and who gets the chance to discover it.

### 3.9 Implementation considerations

Translating AI-enhanced evaluation from concept to practice requires careful attention to institutional readiness and methodological rigor. The most promising near-term approach involves controlled experimentation within existing funding frameworks rather than wholesale system replacement. Funding agencies could begin by implementing parallel review processes–simultaneously evaluating matched proposal pools through both traditional panels and AI-augmented systems–to generate empirical evidence about comparative effectiveness, bias reduction, and outcome quality.

Such trials would need to address several critical implementation challenges. Technical infrastructure must be developed with appropriate safeguards for researcher privacy and institutional compliance, while evaluation metrics should extend beyond traditional citation counts to include measures of innovation, interdisciplinary impact, and long-term field transformation. Equally important is the cultivation of institutional culture change, as successful implementation requires buy-in from both reviewers and applicants who may initially resist algorithmic evaluation.

The transition period offers an opportunity to iteratively refine AI systems based on real-world performance rather than theoretical assumptions. This empirical approach–testing, measuring, and adjusting–represents a more scientifically rigorous pathway than immediate full-scale deployment. Moreover, such controlled trials could provide the evidence base necessary to convince traditionally conservative funding institutions that AI-enhanced evaluation serves scientific progress rather than merely technological novelty.

## 4 The youth revolution: how AI will transform scientific careers

Scientific progress has depended on boldness at the edge of consensus—but in today's academic funding system, boldness is often delayed. Early-career scientists, historically responsible for many of science's most transformative insights, are now forced to wait. Structural biases in funding systems have steadily shifted the arc of scientific independence later into researchers' careers, narrowing the window for risk-taking and innovation. The integration of artificial intelligence into research evaluation offers a rare opportunity to reverse this trend. By redesigning the very systems that allocate resources and recognition, AI-enhanced evaluation could reinvigorate scientific careers—and accelerate discovery itself.

AI systems can be designed to address the deeply embedded biases that currently disadvantage early-career researchers and reverse the age trend in scientific funding. Traditional peer review overweights past performance and underweights future potential through systematic bias (Hofstra et al., 2020). AI systems, by contrast, can focus on proposal quality rather than researcher pedigree, opening doors for early-career investigators. Furthermore, younger scientists, less invested in prevailing paradigms, are more likely to propose truly novel combinations of ideas (Fortunato et al., 2018). AI systems designed to detect conceptual novelty could help surface this often-overlooked innovation potential. Additionally, current systems heavily reward institutional familiarity and professional networks (Li and Agha, 2015), but AI-assisted evaluation can minimize these network effects, helping to level the playing field for newcomers. Reducing these biases could significantly lower the average age of first major grant receipt—by 5 to 7 years—better aligning funding with peak creative periods. In doing so, AI could help restore the natural rhythm of scientific contribution: one where early brilliance is not deferred until it is safe, but nurtured when it is bold.

Current funding systems concentrate resources in elite institutions through both formal mechanisms and unspoken norms (Wahls, 2018), but AI-enhanced evaluation can help democratize access across institutions and decentralize this imbalance. AI can remove institutional identifiers from first-pass assessments through institution-blind evaluation, preventing prestige bias from distorting reviewer judgment (Li and Agha, 2015). Machine learning models can adjust for institutional infrastructure through equipment-normalized expectations, reducing unfair penalties for researchers with fewer resources (Wahls, 2018). Additionally, AI-enabled portfolio optimization could support regionally and institutionally diverse funding ecosystems through geographic and institutional portfolio diversity, tapping into a broader base of intellectual potential. This democratization is not only a matter of fairness—it is a strategy for accelerating discovery.

The cumulative effect of these changes would be nothing short of transformative for accelerating scientific careers and discoveries, fundamentally altering both the pace and structural trajectory of scientific advancement. AI-enhanced evaluation systems could empower younger scientists to lead ambitious research programs nearly a decade earlier than current norms by enabling earlier and more sustained research independence. Even a one-year reduction in the average age of independence could yield a 5–8% increase in lifetime scientific productivity (see Jones, 2009). Freed from the need to appease senior gatekeepers through reduced loyalty signaling, early-career researchers could pursue more independent and unconventional lines of inquiry (Azoulay et al., 2011). With evaluation focused on ideas rather than orthodoxy, scientists would be freer to challenge prevailing paradigms through more diverse research approaches—precisely the conditions under which major discoveries tend to emerge (Foster et al., 2015). AI-enhanced systems could thus unlock not only earlier independence but also more creative and self-directed scientific lives. By restructuring who gets to take risks and when, these reforms could expand the horizon of what science is willing—and institutionally able—to imagine.

In sum, artificial intelligence is not just a means for optimizing review—it is a catalyst for reimagining scientific careers. By shifting the locus of opportunity earlier in the pipeline and broadening access across institutions, AI could help science return to its most generative rhythm: one that rewards imagination over conformity, and possibility over pedigree. This would not simply change who receives funding—it would change what kinds of ideas enter the canon of science in the first place.

## 5 Beyond gatekeeping: a new scientific future

The introduction of AI-enhanced evaluation represents more than a technical upgrade to the grant review process—it marks a fundamental shift in how science defines merit, allocates resources, and determines who is invited to participate in the project of discovery. Whereas current systems often filter out novelty under the guise of rigor, AI offers the potential for a more expansive, evidence-based, and inclusive vision of scientific possibility.

Conventional funding systems operate primarily as gatekeepers—identifying flaws, enforcing norms, and maintaining intellectual boundaries. AI-enhanced approaches could invert this logic, shifting from exclusion to cultivation by seeking promise rather than flaws. While human reviewers are trained to identify reasons to reject, AI systems could be explicitly designed to identify elements of novelty, potential, and unorthodox insight that merit investment. These systems could expand rather than restrict the scope of scientific inquiry, continuously expanding the pool of viable researchers by surfacing promising work from beyond established institutions and disciplinary silos, where existing review structures tend to reinforce closed networks and elite circles. Moreover, algorithmic systems can be designed to evolve in response to empirical outcomes, creating a learning system that improves over time, whereas human review cultures often rely on precedent and inertia. This shift from gatekeeping to opportunity creation could reorient scientific culture itself—from one that rewards conformity and credentialism to one that actively seeks out risk, difference, and intellectual diversity.

A truly innovative funding ecosystem would not be dominated by a single evaluative mechanism, but would instead feature multiple, parallel approaches tailored to different types of inquiry. This diversified landscape could include AI-optimized traditional grants that enhance project-specific funding through algorithmic triage and bias correction, investigator-based models following HHMI-style approaches that fund people rather than projects to enable sustained creative independence, and challenge-based allocation through prizes, competitions, and milestone-driven awards aligned with concrete scientific goals. Market-based mechanisms such as science prediction markets or crowd-based evaluation models could harness collective intelligence, while AI-enabled microgrants could provide small, rapid-turnaround funds to support early-stage exploration with minimal administrative burden. This diversification would distribute power, encourage experimentation, and create natural laboratories for testing what works most effectively.

One of the most transformative promises of AI-enhanced funding lies in its ability to learn from itself. Unlike legacy systems that operate without feedback or accountability, AI models can be continuously updated based on real-world outcomes through comprehensive outcome tracking that assesses funded proposals across multiple time horizons and metrics—citations, replication, translational impact—to evaluate program effectiveness. Counterfactual analysis could randomly fund a portion of initially rejected proposals to identify false negatives and calibrate reviewer and algorithmic performance, while system evolution would allow evaluation models to be updated regularly, refining scoring functions, bias detectors, and novelty detection based on empirical evidence. While peer review is often mythologized as the “gold standard” of scientific evaluation, it remains largely unevaluated by the standards of science itself (Smith, 2006). AI-enhanced systems offer a rare opportunity to turn evaluation into an evidence-generating process—not just for research outcomes, but for the system that chooses them.

The contrast between youth-driven innovation in AI and the increasingly gerontocratic funding patterns in the biological sciences reveals how institutional structures shape scientific progress. Traditional study section models of peer review have evolved into self-reinforcing systems that favor established researchers pursuing incremental advances while systematically excluding early-career scientists who offer potentially transformative ideas. In a profound irony, artificial intelligence—a field that continues to empower early-career innovators and reward unconventional thinking—now offers a radical alternative for how research funding could be structured across scientific disciplines. AI-enhanced evaluation systems could reduce entrenched bias, open doors to broader participation, and accelerate the pace of discovery by disrupting the deeply rooted hierarchies that govern resource allocation. This transformation could create new, more equitable pathways to innovation, level the playing field for those outside elite networks, and help revive a spirit of boldness in a system increasingly constrained by caution.

The stakes extend well beyond academic careers to society's ability to meet global challenges. The current system favors those who can guarantee results rather than those with potentially transformative ideas that, by definition, cannot promise certainty of success (Alberts et al., 2014). This bias against uncertainty—and thus against innovation—undermines science precisely when humanity faces complex, urgent crises. From climate change and pandemic disease to ecological collapse, dwindling resources, and the growing burden of chronic illness, neurodivergence, and psychological stress, the problems of our time demand not just new answers but entirely new ways of thinking. These challenges are not only scientific but deeply human, requiring systems that value creativity, intellectual risk, and the capacity to imagine what does not yet exist.

By dismantling the study section stranglehold, AI could help unlock the full creative potential of the scientific enterprise. Early-career researchers could pursue bold, unconventional projects without spending decades navigating institutional bottlenecks, while scholars from historically underfunded or marginalized institutions could finally compete on more equal footing. Truly novel approaches could receive support based on intrinsic promise rather than proximity to established paradigms, restoring a sense of scientific possibility and intellectual risk-taking often lost in the grind of grantmanship, careerism, and incrementalism. The promise is real, but so are the risks, and AI-driven systems must themselves be designed with transparency and accountability to avoid reproducing the very exclusions they aim to overcome.

In the long arc of scientific reform, AI may prove to be less an instrument than a turning point. It invites us to imagine a future in which scientific promise is judged not by pedigree, proximity, or prestige, but by the quality of ideas and the breadth of possibilities. This transformation would not be merely technical but deeply human, replacing gatekeeping with opportunity, institutional inertia with imagination, and systemic exclusion with inclusion. Science advances fastest when it includes diverse perspectives, approaches, and participants, yet our current funding systems systematically exclude precisely this diversity. Addressing this exclusion represents perhaps the single greatest opportunity to accelerate scientific progress. Artificial intelligence offers a viable path toward a more open, generative, and forward-looking scientific future where researchers of all ages, disciplines, and institutions could compete based on the strength of their ideas rather than the prestige of their pedigrees. The very technology that has reshaped countless other domains may now be poised to transform science itself—helping to unlock human potential and accelerate discovery when the world needs it most. This is not a call to abandon human judgment, but to re-engineer it—by embedding it within systems that are transparent, accountable, and capable of learning. We may finally realize a scientific funding system that does not merely reward those who fit the mold but invests in those bold enough to reshape it. The stakes are not just procedural—what we choose to fund today determines what we will be able to understand tomorrow. The future of science may depend not just on what we discover—but on how we decide who gets to try.

While senior scientists and other beneficiaries of the current system may continue to advocate for traditional study sections, the empirical evidence supporting their effectiveness remains limited and contested. Numerous evaluations of peer review have revealed persistent issues, including inconsistent scoring, susceptibility to both conscious and unconscious bias, and low inter-rater reliability across panels. To rigorously assess the potential of alternative models, funding agencies could initiate controlled, large-scale comparisons—for instance, allocating matched pools of proposals through both conventional panels and AI-assisted review systems. Resulting outcomes could be analyzed not only in terms of demographic characteristics of awardees (such as age, institutional affiliation, discipline, and career stage), but also with respect to the long-term scientific impact, innovation potential, and productivity generated by the funded research. Such trials would provide a more robust, evidence-based foundation for evaluating the comparative fairness, efficiency, and effectiveness of competing research funding mechanisms.

## Data Availability

The original contributions presented in the study are included in the article/supplementary material, further inquiries can be directed to the corresponding author.
